# A community effort to assess and improve computerized interpretation of 12-lead resting electrocardiogram

**DOI:** 10.1007/s11517-021-02420-z

**Published:** 2021-10-22

**Authors:** Zijian Ding, Guijin Wang, Huazhong Yang, Ping Zhang, Dapeng Fu, Zhen Yang, Xinkang Wang, Xia Wang, Zhourui Xia, Chiming Zhang, Wenjie Cai, Binhang Yuan, Dongya Jia, Bo Chen, Chengbin Huang, Jing Zhang, Yi Li, Shan Yang, Runnan He

**Affiliations:** 1 Department of Electronic Engineering, Tsinghua University, Beijing, China; 2 Department of Cardiology, Beijing Tsinghua Changgung Hospital, Beijing, China; 3 School of clinical Medicine, Tsinghua University, Beijing, China; 4 Chinese Academy of Sciences Zhong Guan Cun Hospital, Beijing, China; 5 ECG Center, Tianjin Wuqing District People’s Hospital, Tianjin, China; 6 ECG Diagnosis Department, Fujian Provincial Hospital, Fuzhou, China; 7 Beijing Tsingdata Technology Development Co., LTD., Beijing, China; 8 Tsinghua-Berkerley Shenzhen Institute, Shenzhen, China; 9 Southwest University of Science and Technology, Mianyang, China; 10 University of Shanghai for Science and Technology, Shanghai, China; 11 Rice University, Houston, USA; 12 Guangzhou Shiyuan Electronic Technology Company LTD, Guangzhou, China; 13 1st Military Delegate Room of Dalian Regional, Dalian, China; 14 East China Normal University, Shanghai, China; 15 University of Science and Technology of China, Hefei, China; 16 China Wuhan Zoncare, LTD., Wuhan, China; 17 Chengdu Spaceon Electronics CO., LTD., Chengdu, China; 18 Harbin Institute of Technology, Harbin, China

**Keywords:** Electrocardiogram, Computersized interpretation of electrocardiogram, Model assessment, Deep neural networks

## Abstract

Computerized interpretation of electrocardiogram plays an important role in daily cardiovascular healthcare. However, inaccurate interpretations lead to misdiagnoses and delay proper treatments. In this work, we built a high-quality Chinese 12-lead resting electrocardiogram dataset with 15,357 records, and called for a community effort to improve the performances of CIE through the China ECG AI Contest 2019. This dataset covers most types of ECG interpretations, including the normal type, 8 common abnormal types, and the other type which includes both uncommon abnormal and noise signals. Based on the Contest, we systematically assessed and analyzed a set of top-performing methods, most of which are deep neural networks, with both their commonalities and characteristics. This study establishes the benchmarks for computerized interpretation of 12-lead resting electrocardiogram and provides insights for the development of new methods.

**Supplementary information:**

The online version contains supplementary material available at 10.1007/s11517-021-02420-z.

**Graphical Abstract:**
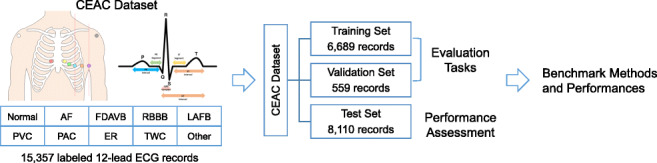
A community effort to assess and improve computerized interpretation of 12-lead resting electrocardiogram

## Introduction

Cardiovascular disease is the leading cause of death around the globe [25] and becomes a heavy burden in the world’s largest population—China [20]. Electrocardiogram (ECG) is essential to diagnose and screen cardiovascular diseases (CADs) including arrhythmia, myocardial infarction, and hypertrophy. It is one of the most common procedures in daily cardiovascular healthcare, with 3 million ECGs estimated to be performed worldwide every day [27]. However, about 20 percent of CIE is incorrect based on a rough estimation [22], and unrecognized mistakes are more likely to result in misdiagnoses and delay the proper treatments [26]. Therefore, improving CIE help lay the foundation for the precision diagnosis of CADs, leading to better cardiovascular healthcare.

High-quality ECG data helps promote the development of CIE. Most previous studies are based on the MIT-BIH Arrhythmia Database, which consists of 2-lead Holter data monitored from 48 patients [24]. Though these 48 ECG records were carefully annotated, standard 12-lead ECGs have become the mainstream in clinical practice. The Common Standards for Electrocardiography database, containing 1,000 standard 12-lead resting ECG records, is applied to assess for wave delineation since the late 1980s [31]. More standard 12-lead ECG datasets are published later, such as the Physionet 2011 challenge dataset for signal quality evaluation and the STAFF III dataset for coronary artery identification [23, 32]. The CPSC2018 dataset provides about 9 thousand 12-lead resting ECGs with nine types of interpretations, which however take only a small fraction among various clinical interpretations [19]. Though various methods showed their efficiencies on these datasets with limited patient samples [3, 30, 33], lacking disease patterns in a larger population hinders algorithm developing and performance assessment.

Deep neural networks are promising to play an important role in the daily clinical practice of ECG monitoring and interpretation [16]. For example, a 34-layer convolutional neural network was reported to outperform ECG technicians on single-lead Holter data [9]. Physionet Challenge 2017 offers a chance for the research community to compete on atrial fibrillation prediction based on short-duration single-lead data [4]. Three of the four winning teams utilized deep neural networks combined with handcrafted expert features [5, 12, 28]. However, these single-lead ECG records were recorded by wearables and cannot provide as much information as standard 12-lead ECGs.

A large volume of 12-lead ECG data with high-quality interpretations is crucial to assess the deep learning based CIE. Though CPSC2018 made the first attempt in China [19], its criteria for assessment ignores the fact that a record may contain more than one abnormality. Therefore, there is urgent need for a better understanding that how much machine learning methods, especially deep neural networks, can improve the predictive performance for standard 12-lead ECG data. However, to our knowledge, there are no previous studies that systematically assess and analyze a set of algorithms based on a common dataset.

In this paper, we report a novel dataset consisting of 15 thousand 12-lead resting ECG records, as well as a systematic assessment and analysis of benchmark algorithms from the China ECG AI Contest (CEAC) 2019 [1]. This dataset covers most types of clinical interpretations revised by four doctors and reflects the multi-label characteristics in clinical practice. Based on this novel dataset, CEAC 2019 calls for a community effort to assess and improve the computerized interpretation of 12-lead ECG.

We analyzed the top-performing methods, most of which are deep neural networks, aiming to identify successful cases. Our findings mainly include four aspects: (1) the network structure composed of CNN, RNN and attention can achieve excellent predictive performances; (2) incorporating external information, such as learning from other data or expert knowledge, can alleviate the overfitting problem; (3) data augmentation, focal loss and weighted cross-entropy are effective for imbalanced data; (4) multi-task learning and post-processing are utilized to deal with the multi-label classification problem. This systematic analysis may provide insights for future researches.

## The CEAC dataset and evaluation tasks

An ECG records the electrical activities in the heart, and a 12-lead resting ECG is a common examination in the clinic to diagnose arrhythmia, myocardial infarction, and hypertrophy. We built a novel dataset consisting of about 15 thousand 12-lead resting ECG records, to train, validate and test different algorithms from both academia and industry. Since CEAC 2019 calls for a community effort to improve CIE, this dataset is defined as CEAC Dataset V1.0, which will be added with more data and more careful annotations in the future. To our knowledge, this is currently the largest dataset with a 60 percent increase compared to the state-of-the-art dataset [19].

There are mainly three points that distinguish the CEAC dataset from others:

1. it is currently the largest standard 12-lead ECG dataset in China to our knowledge;
2. it covers most types of clinical interpretations revised by doctors and technicians;
3. it reflects the fact that one ECG record may contain more than one abnormality.

This dataset provides the training, validation, and test set with the same statistical characteristics for assessing different algorithms. Researchers are welcome to have access to the CEAC dataset by contact with the corresponding author through the website [2].

### Dataset building

All ECG records were collected from four hospitals in China. Four experts focused their time and efforts to annotate and review all ECG samples. To make the interpretations as correct as possible, two doctors and two technicians made up two teams, with each team consisting of one doctor and one technician. The workflow of annotating and reviewing is the same as in clinical practice, with one technician annotating an ECG record, and one doctor reviewing this record. The experts utilized a web-based tool for distributed ECG annotation in a local area network [7]. The dataset building has been approved by the ethics committees of the four hospitals.

### Basic statistics

The complete dataset consists of 15,357 records. We select labels with as many samples as possible to represent the interpretations, resulting in 10 labels including normal ECGs (Normal), atrial fibrillation (AF), first-degree atrioventricular node block (FDAVB), right bundle branch block (RBBB), left anterior fascicular block (LAFB), premature ventricular contractions (PVC), premature atrial contraction (PAC), early repolarization (ER), T wave change (TWC) and other ECGs (Others). The clinical definitions of each label are summarized in the Supplementary Materials. In short, the first 9 labels refer to normal ECGs and those with abnormalities, and ‘Others’ refers to those records which cannot be exactly descript by any of the 9 types. Since many abnormalities are relatively rare according to daily practices in the clinic, we gathered these types in one type ‘others’ such as atrial flutter and pre-excitation. As a result, compared to the latest 12-lead resting ECG dataset [19], the CEAC dataset covers most interpretation types.

**Fig. 1:**
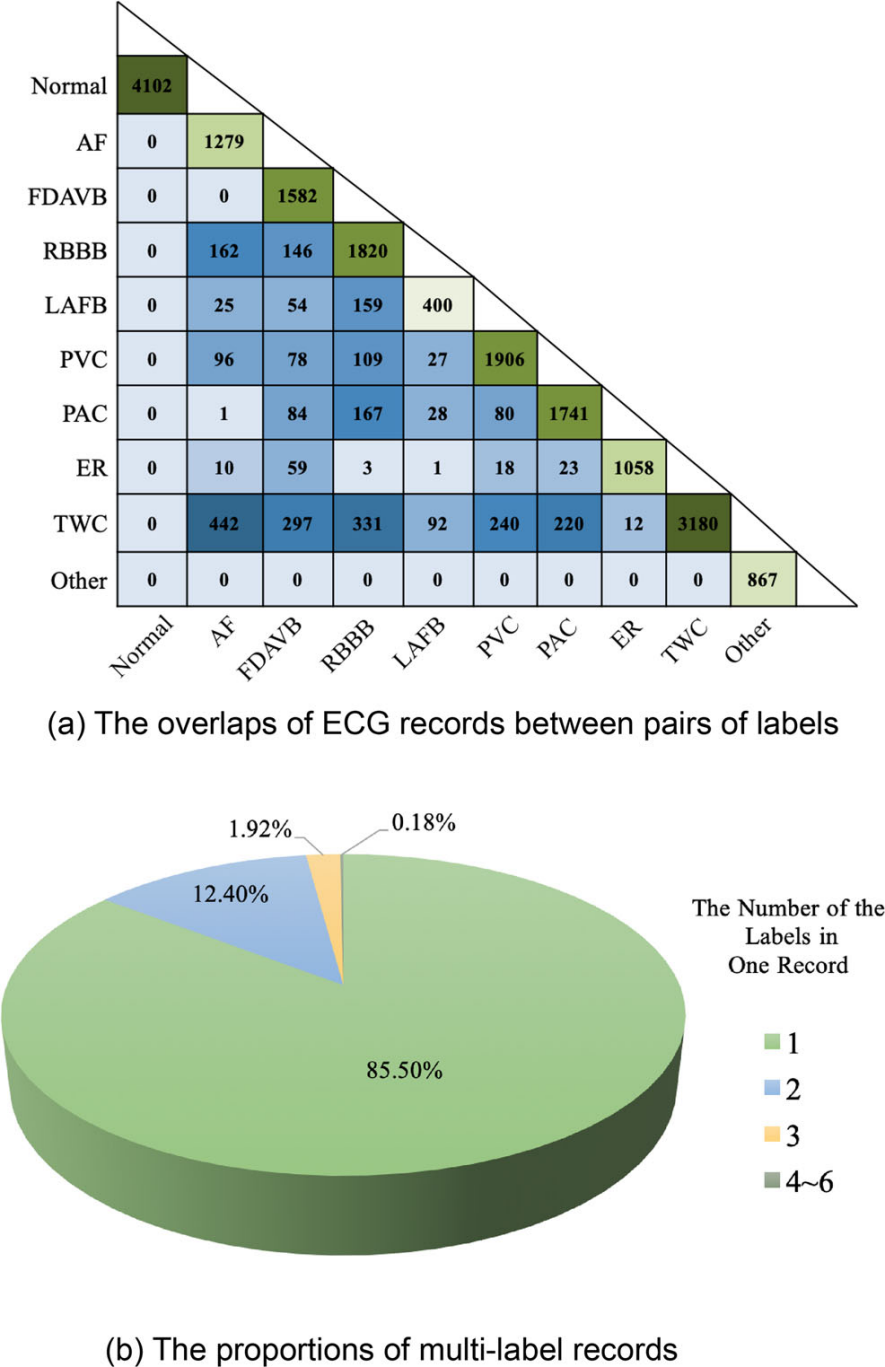
Multi-label clinical interpretations of all ECG records in the CEAC dataset. (a) reflects the multi-label characters among each pair of clinical interpretations. (b) shows that almost 15 percents of all records contain more than one interpretations

The interpretations of ECG records are shown in Fig. 1. In Fig. 1(a), the darker green boxes represent the larger numbers of samples. The samples labeled as Normal, TWC, and PVC are the top three, while LAFB, Others, and ER are the bottom three. Since one ECG record may contain more than one abnormality, Fig. 1(a) also shows the co-existence for every pair of labels. The darker blue boxes represent the more frequent pairs. For example, AF is more often to co-exist with TWC and RBBB. The lighter blue boxes represent the less frequent pairs. For example, normal never co-exist with other labels; Others never co-exist with any other nine labels; AF never co-exists with FDAVB. The proportions of multi-label records are shown in Fig. 1(b). The number of multi-label samples takes up no more than 15 percent in total. Among these samples, the majority have two labels. The samples with more than four labels take up less than 1 percent.

**Fig. 2:**
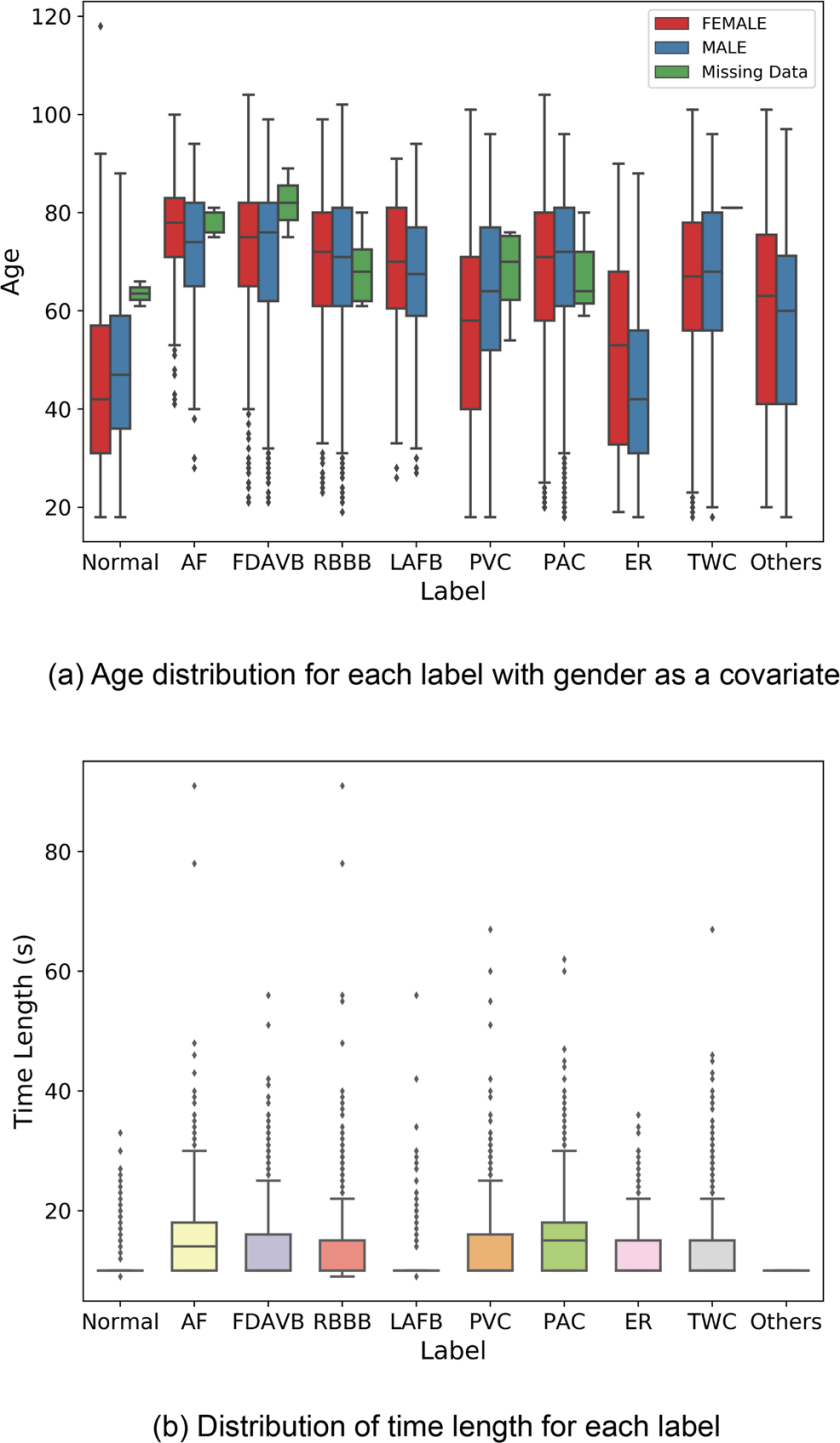
Basic statistics of all records in the CEAC dataset. (a) shows the age distributions among each clinical interpretation, (b) shows that the distribution of time length of all records. The error bars are percentiles

The clinical variables including age and gender are shown in Fig. 2. Figure 2(a) shows the age distribution under each label, with gender as the covariate. Since some records have no gender information, Fig. 2(a) represents them as missing data. Patients labeled as Normal is relatively younger than most of the other 9 labels since the elderly are more likely to have cardiovascular diseases. Male patients with ER are the second youngest than the others except for Normal. This suggests that both age and gender can be a feature to predict ER. As shown in Fig. 2(b), though most samples are recorded for 10 seconds, the time length varies across all records.

To assess different algorithms, the complete dataset is divided into the training set with 6,689 records, validation set with 559 records, and test set with 8110 records with similar statistical characteristics. All algorithms can be trained and validated on the training and validation set. The test set remains private to assess or evaluate the generalization ability.

### Evaluation tasks

CEAC 2019 aims to call for a community effort to evaluate the current state of computerized interpretation of 12-lead resting ECGs, to set up the benchmark predictive performances, and to provide insights for further research. Three rounds of the contest, including a preliminary, a rematch and a final, were set to gradually screen competitive participating teams.

During the three rounds of the contest, we set up three evaluation tasks respectively: (1) how well do algorithms distinct abnormal ECGs from normal ones? (2) how well do algorithms predict the eight abnormalities or Normal for one ECG record? (3) how well do algorithms predict a record that falls into none of the nine pre-defined categories, namely the Others? In the preliminary, we screened the top 100 among the 354 participating teams; in the rematch, we screened the top 23 teams among the 68 valid submissions; in the final, we received 21 valid submissions.

In this paper, we discuss the third task set up for the final of the contest. Because this is the most complete task that is closely related to clinical practices, and also requires the complete dataset to develop and assess algorithms.

There are several challenges in the final evaluation task as follows:

- how to efficiently extract features from data with variable time lengths;
- how to overcome the overfitting problem, which is quite usual to deep neural networks;
- the number of samples varies among different labels. Imbalanced data often leads to overfitting on labels with more data [10];
- one record may contain more than one abnormality, thus a multi-label classification problem needs to be solved.

In addition, all participating teams faced a common difficulty in that there was no glance at the hidden test set. All developing and training procedures should be accomplished based on the training and validation set.

### The scoring metrics

To assess the predictive performances, we use the measurements based on multi-label classification [37]. For each of the category 1 ≤ *j* ≤ 10 and each of the ECG record 1 ≤ *i* ≤ *N*, there are four quantities to measure predictive results.

1 $$ TP_{j} = |{x_{i}|y_{j} \in Y_{i}, y_{j} \in f(x_{i}), 1\leq i \leq N}| $$

2 $$ FP_{j} = |{x_{i}|y_{j} \not\in Y_{i}, y_{j} \in f(x_{i}), 1\leq i \leq N}| $$

3 $$ TN_{j} = |{x_{i}|y_{j} \not\in Y_{i}, y_{j} \not\in f(x_{i}), 1\leq i \leq N}| $$

4 $$ FN_{j} = |{x_{i}|y_{j} \in Y_{i}, y_{j} \not\in f(x_{i}), 1\leq i \leq N}| $$

Based on the four above quantities, we can define precision, recall and *F*_1_ score for each category,

5 $$ Precision_{j} = \frac{TP_{j}}{TP_{j} + FP_{j}} $$

6 $$ Recall_{j} = \frac{TP_{j}}{TP_{j} + FN_{j}} $$

7 $$ F_{1j} = \frac{2 \cdot Precision_{j} \cdot Recall_{j}}{Precision_{j} + Recall_{j}} $$

The final *F*_1_ score for each team is the average of each category.

## Benchmark methods and performances

**Fig. 3:**
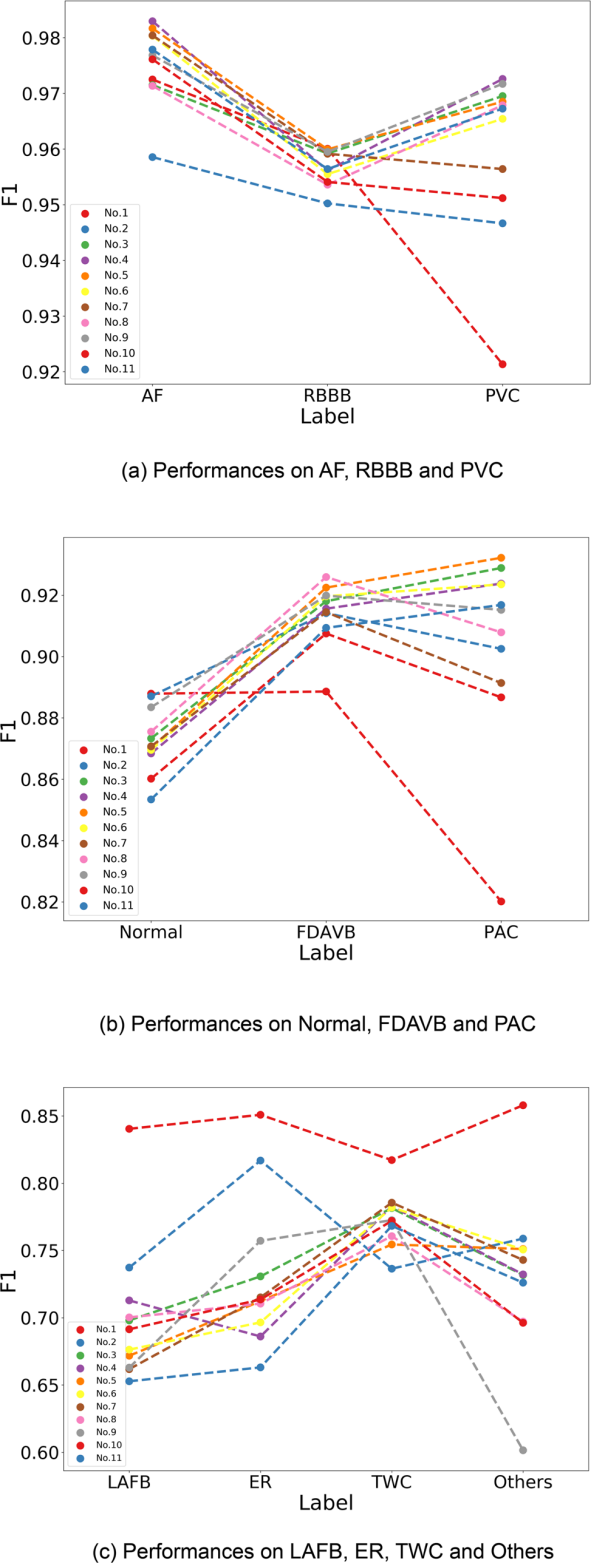
Assessing the *F*_1_ scores of the top 11 methods. (a) shows the three interpretations with the highest average scores, (b) shows three with the modest scores and (c) shows the lowest

Aiming to identify success cases and to provide insights for further research, we analyzed the top 11 out of the 21 methods in the final of CEAC 2019, most of which are deep neural networks. We summarized their properties in the view of supervised learning. Table 1 lists the methods with some of their key properties and the final *F*_1_ scores. Figure 3 shows the *F*_1_ scores of each method on each label. We also calculated the accuracies of each method as in Table 1 in the Supplementary File.

**Table 1:**
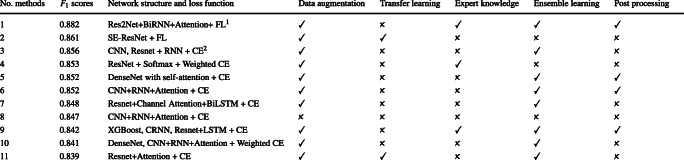
Summary of the Top-Performing 11 Benchmark Methods. All methods are ranked according to their *F*1 scores. The network structures are summarized and their characteristics are shown as in data augmentataion and transfer learning, etc

^1^FL refers to focal loss. ^2^CE refers to cross entropy

To summarize how the top methods deal with the four challenges mentioned above, key properties are grouped into data preprocessing, feature engineering, and classifiers. In data processing, it is shown that data alignment is necessary to cope with various time lengths (Challenge 1). In feature engineering, the first part summarizes how to design network structures to efficiently extract features; the second part summarizes how to apply external information to overcome the overfitting problem (Challenge 2). In the design of classifiers, focal loss and weighted cross-entropy are found to perform excellent among the top methods (Challenge 3); multi-task learning and postprocessing are utilized for multi-label classification (Challenge 4).

### Data preprocessing

The main purpose of data preprocessing is to provide samples that are suitable for feature engineering. Considering the characteristics of 12-lead resting ECG data, researchers need to design strategies to cope with signals of various time lengths and improve signal qualities.

#### Signal processing

is utilized to improve signal qualities. Since the unit of ECG signals is millivolts, and ECG is often contaminated with noises such as baseline wander, muscle artifact and electrode motion artifact, etc., denoising is a key step to improve signal-to-noise ratios.

#### Data alignment

The ECG records of the CEAC dataset vary in time lengths, as shown in Fig. 2(b). Deep neural networks such as CNNs usually require a fixed input size of data for feature learning. Therefore, appropriate processing strategies are essential to align all ECG records to an equal length.

Padding and cropping are applied for data alignment. For short signals, padding to either side helps to fix time lengths. One strategy is to pad with zeros, which adds no information and can be handled by convolutions. Another strategy is to pad with self-repeated signals, which adds repetitive information.

Long signals are cropped into multiple segments with or without overlapping windows. These segments are labeled after the original long signals. However, for isolated abnormalities such as PVC and PAC, some segments without them are also labeled as PAC or PVC, which result in incorrectly labeled samples. To deal with this disadvantage, Method 3 manually labels all PAC and PVC segments; Method 4 applies a heuristic strategy to filter segments unlikely to be PVC or PAC. As a result, both methods achieved high *F*_1_ scores in PVC and PAC.

#### Data augmentation

Cropping one signal to several segments can also be seen as a data augmentation strategy. Long signals belonging to labels with fewer samples can be augmented, which may help deal with the imbalanced data problem. Up-sampling with replacement is also applied to overcome the ignorance of those labels with fewer data. Instead of directly up-sampling, some methods multiply the signals with a random coefficient closed to 1. Some methods also down-sample normal and TWC samples, which have the largest data sizes according to Fig. 1(a). Both up-sampling and down-sampling help alleviate overfitting on labels with more data.

Method 2 converts the 1D time-series data to 2D images by plotting the signals as curves on a fixed background, therefore transforming the original task into a computer vision task. However, several methods also utilize this strategy but never achieved as high *F*_1_ scores. An important trick is to color the signal curves on the images, and different color combinations affect predictive performances on both the training and validation set. As for data augmentation, Method 2 finds that affine transformation can improve predictive performances, while other traditional image processing procedures like flipping, lighting or rotating decrease the *F*_1_ scores.

### Feature engineering

Feature engineering here refers to extracting useful features that can represent the key ECG characteristics of different labels. In this section, the first part mainly summarizes efficient strategies to design deep neural networks; the second part mainly summarizes how to incorporate external information and overcome the overfitting problem.

#### Design of deep neural networks

**Fig. 4:**
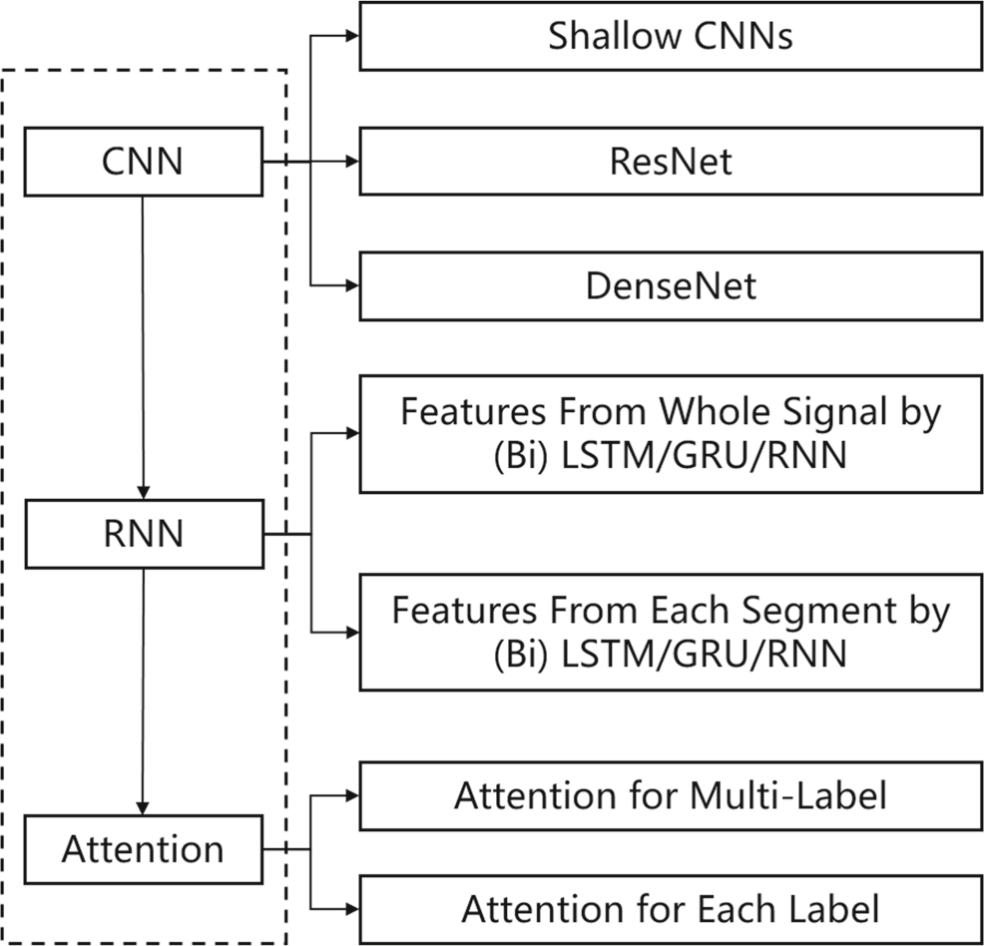
The commonalities of all top-performing deep neural networks. CNN layers combined with RNN layers and attention modules can achieve good performances

It is found that a common network structure is utilized and proved its efficiency based on the high *F*_1_ scores. As shown in Fig. 4, this common structure is composed of convolutional layers, recurrent layers, and attention modules. This network structure is reasonable to analyze time-series data such as ECG signals [34]. Firstly, the convolutional layers extract features and reduce dimensions. Deeper features with fewer dimensions generally represent data at a more abstract level [17]. Secondly, the recurrent layers, usually bidirectional RNN or LSTM, learn the correlations among the deep features. This is suitable for ECG signals in that time dependencies are represented in the P-QRS-T waves. Thirdly, the attention modules, which allow modeling of dependencies without regard to the distance in the input sequences [29], can give higher weights to features that are correlated to a specific label.

Other methods among the top 11 also follow this structure, but lacking either the recurrent layers or the attention modules. For example, Method 2 transforms to an image classification problem and therefore ignores the recurrent layers; Method 4 applies a 1D ResNet [35] and ignores both recurrent layers and attention modules; Method 5 utilizes a 1D DenseNet and self-attention. These methods ignore the time dependencies and focus more on the shapes of ECG signals.

##### The backbone CNNs

are essential to extract features from ECG signals. More powerful feature extraction is more likely to achieve higher *F*_1_ scores. In Table 1, both method 6 and method 8 apply a relatively shallow backbone consisting of 15 convolutional layers and achieve *F*_1_ scores close to other more complex structures. Their high performances suggest that most ECG features can be captured by relatively shallow backbone CNNs.

According to Table 1, 6 out of 11 methods apply residual blocks, including Res2Net [8], SE-Resnet [13] and different versions of ResNet [11], as shown in Fig. 4. Stacking more residual blocks to form deeper CNNs enhances feature representation abilities and increases predictive performances [11]. Method 1 applies Res2Net to promote multi-scale representation ability [8]; Method 2 applies SE-ResNet to capture the channel-wise relationships [13]. Besides ResNet, DenseNet is applied by 2 methods [14]. With the increase of the CEAC data size in the future, these deeper networks may develop stronger capacities and capabilities.

##### The recurrent layers

are essential to explore the dependencies among features representing ECG signals. Simple RNN, LSTM, GRU and their bidirectional versions are applied by 7 methods as shown in Table 1. These applications of recurrent layers can be roughly divided into two types. One type is to learn the correlations among features of one segment; the other is to learn the correlations among features from several segments, as shown in Fig. 4.

##### The attention modules

give different weights to different features. According to Table 1, 8 methods utilize various types of attention modules, which can be grouped into three types of strategies, as shown in Fig. 4. The first one is to weigh different features output by the recurrent layers. The second one is to apply one attention module to each label [21]. The last one is to combine with the backbone CNNs, such as the squeeze-and-excitation layers combined with ResNet in Method 2 [13] and the self-attention combined with DenseNet in Method 5. Attention modules are supposed to be effective for predicting labels with isolated events, including PVC and PAC. From Fig. 3(a) and (b), methods with attention modules often achieve high *F*_1_ scores on these two labels.

#### Incorporating external information

**Fig. 5:**
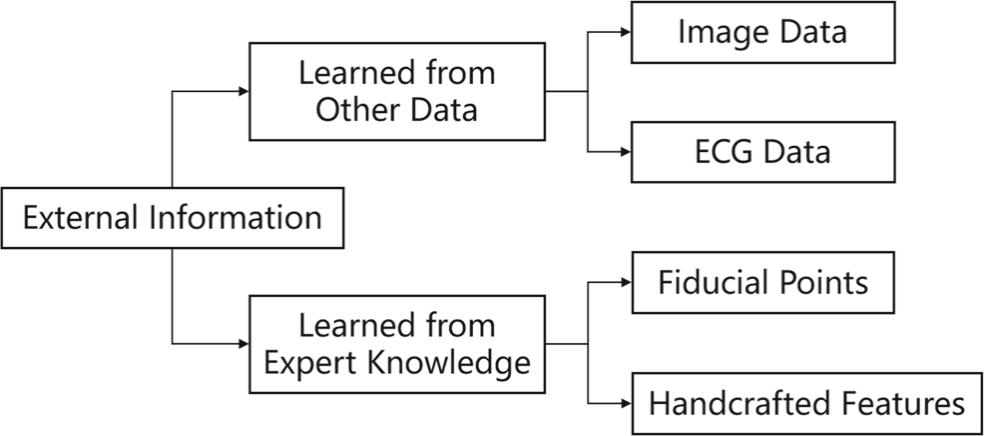
Incorporating external information is one way to alleviate the overfitting problem common to deep neural networks. Some top-performing methods either learning knowledge from other dataset or from expert knowledge

Incorporating more information is effective to overcome the overfitting problem. For example, learning from other data or transfer learning can pretrain networks by external datasets and therefore incorporate information from these datasets; learning from expert knowledge can also improve predictive performances by introducing inductive bias. According to Fig. 5, several top methods are summarized and grouped into either learning from other data or from expert knowledge.

##### Learning from other data

or transfer learning refers to modeling a neural network on a different but somehow similar problem and therefore partially reuse the network parameters to accelerate training and improving performance. Since Method 2 transforms into an image classification problem, it pretrains the SE-ResNet on the ImageNet dataset [6]. Method 11 pretrains its network on the CPSC2018 dataset, whose labels are different from the CEAC dataset.

##### Learning from expert knowlege

to extract handcrafted features can assist neural networks to improve performances. Three methods apply handcrafted features in different ways. Method 1 identifies fiducial points of P-QRS-T waves on each of the leads, then inputs this information to a deep neural network for automatic feature extraction [38]. Method 4 first identifies the R peaks and then calculates statistical features such as RR intervals and QRS wave widths. Instead of inputting these features to a neural network, Method 4 combines the handcrafted features with deep features extracted by the backbone ResNet [35]. Method 9 extracts various types of handcrafted features related to LAFB and ER and inputs them to an XGBoost.

### Classifiers

All methods need to predict multi-labels for each ECG record. According to Fig. 1(b), about 15 percent of all records are labeled with more than one abnormality. In this section, it is shown that all top 11 methods apply multi-task learning to make multi-label predictions. Due to the imbalanced data problem, these methods also need to find proper loss functions. Also, almost all methods apply ensemble learning to improve accuracy and postprocessing to make more reasonable predictions due to some known relationships among different labels.

#### Multi-task learning

treats each label prediction as a separate task and solves all tasks simultaneously. One of its benefits is to exploit commonalities across different tasks, which leads to smaller model sizes and better performances. According to Table 1, all top 11 methods use multi-task learning to design their networks, in which the decision layers are composed of multiple sigmoid functions. This strategy defines each label prediction as a binary classification task [15]. A positive prediction means the record belongs to one label, while a negative prediction means the opposite. In comparison, some of the methods that are not among the top 11 transform multi-label prediction to several binary classification tasks. This strategy does not share model parameters across different tasks. Modeling correlations among different labels may help improve performances. Method 10 outputs the predicted probabilities of each neural network and input them to an ML-KNN [36].

#### Loss function

plays an important role to deal with imbalanced data according to Table 1. Most methods utilize the weighted binary cross-entropy. It sets a weight coefficient for each label and therefore alleviates overfitting on labels with more data. Both Method 1 and Method 2 apply the focal loss to deal with the class imbalance problem. The standard cross-entropy loss is reshaped such that it down-weights the loss assigned to well-classified samples [18]. In the case of 12-lead resting ECG data, the focal loss results in better performances.

#### Ensemble learning

reduces variances and increases robustness. Since many methods crop long signals into several segments, the summation of corresponding predictions can be either averaging probabilities or majority voting. Some methods also apply bagging to train models on re-sampled datasets.

#### Postprocessing

focuses on the correlations among different labels. The idea is to post-process the results and output more reasonable predictions according to some known relationships. For example, normal ECGs do not co-exist with either abnormalities or Others; AF does not co-exist with FDAVB, etc. The postprocessing strategy can be a good choice when the number of labels is modest. It may become too complex to handle when the number gets too large. Therefore, modeling the correlations among labels can be future directions.

## Conclusion

The building of the largest Chinese 12-lead resting ECG data makes it possible to comprehensively assess different algorithms for CIE. Based on CEAC 2019 [1], we called for a community effort to improve the computerized interpretation of 12-lead resting ECGs. The systematic assessment and analysis of the top-performing deep neural networks establish benchmarks and provide insights for developing new methods. To our knowledge, no previous studies have analyzed a comprehensive set of algorithms based on a common 12-lead resting ECG dataset. We hope these findings might eventually lead to improvements in daily cardiovascular healthcare.

## Electronic supplementary material

Below is the link to the electronic supplementary material.

(PDF 3.88 MB)
