# Supplementary material for: A community effort to assess and improve computerized interpretation of 12-lead resting electrocardiogram
Source: Med Biol Eng Comput. 2021 Oct 22;60(1):33–45. doi: 10.1007/s11517-021-02420-z (PMC8724189; doi:10.1007/s11517-021-02420-z)
Supplement: Supplementary file 1 — (PDF 3.88 MB) [file 11517_2021_2420_MOESM1_ESM.pdf]

Noname manuscript No.
(will be inserted by the editor)

Supplentary Files: A Community Effort to Assess and Improve Computerized Interpretation of 12-lead Resting Electrocardiogram

Zijian Ding[1], Guijin Wang[1], Huazhong Yang[1] Ping Zhang[2,3], Dapeng Fu[4], Zhen Yang[5], Xinkang Wang[6], Xia Wang[7], Zhourui Xia[8], Chiming Zhang[9], Wenjie Cai[10], Binhang Yuan[11], Dongya Jia[12], Bo Chen[13], Chengbin Huang[14], Jing Zhang[15], Yi Li[16], Shan Yang[17] and Runnan He[18]

the date of receipt and acceptance should be inserted later

Abstract This file mainly contains the supplementaries for the paper "A Community Effort to Assess and Improve Computerized Interpretation of 12-lead Resting Electrocardiogram". In this file, the desciptions of clinical definitions for 10 labels of the CEAC dataset are introduced, including normal ECGs(Normal), atrial fibrillation (AF), first-degree atrioventricular node block (FDAVB), right bundle branch block (RBBB), left anterior fascicular block (LAFB), premature ventricular contractions (PVC), premature atrial contraction (PAC), early repolarization (ER), T wave change (TWC) and other ECGs (Others). Since there are many uncommon abnormal types and noise, we only list some of them for explanation.

This work was supported by the The National Key Research and Development Program of China (2017YFB1401804)

Corresponding author: Guijin Wang (e-mail: wangguijin@tsinghua.edu.cn).

[1] Department of Electronic Engineering, Tsinghua University, Beijing, China
[2] Department of Cardiology, Beijing Tsinghua Changgung Hospital,Beijing, China
[3] School of clinical Medicine, Tsinghua University, Beijing, China
[4] Chinese Academy of Sciences Zhong Guan Cun Hospital, Beijing, China
[5] ECG Center, Tianjin Wuqing District People's Hospital, Tianjin, China
[6] ECG diagnosis department, Fujian Provincial Hospital, Fuzhou, China
[7] Beijing Tsingdata Technology Development Co., LTD.,Beijing, China
[8] Tsinghua-Berkerley Shenzhen Institute, Shenzhen, China
[9] Southwest University of Science and Technology, Mianyang, China
[10] University of Shanghai for Science and Technology, Shanghai, China
[11] Rice University, Houston, USA
[12] Guangzhou Shiyuan Electronic Technology Company LTD, Guangzhou, China
[13] 1st Military Delegate Room of Dalian Regional, Dalian, China
[14] East China Normal University, Shanghai, China
[15] University of Science and Technology of China, Hefei, China
[16] China Wuhan Zoncare, LTD., Wuhan, China
[17] Chengdu Spaceon Electronics CO., LTD., Chengdu , China
[18] Harbin Institute of Technology, Harbin, China

1 Definitions of the ten labels

1.1 Normal ECGs (Normal)

As shown Figure 1, sinus rhythm is any cardiac rhythm in which depolarization of the cardiac muscle begins at the sinus node. It is characterized by the presence of correctly oriented P waves on the electrocardiogram (ECG). Sinus rhythm is necessary, but not sufficient, for normal electrical activity within the heart.

1.2 Atrial fibrillation (AF)

As shown Figure 2, atrial fibrillation (AF) is an abnormal heart rhythm characterized by rapid and irregular beating of the atria. Often it starts as brief periods of abnormal beating which become longer and possibly constant over time. Often episodes have no symptoms. Occasionally there may be heart palpitations, fainting, lightheadedness, shortness of breath, or chest pain. The disease is associated with an increased risk of heart failure, dementia, and stroke. It is a type of supraventricular tachycardia.

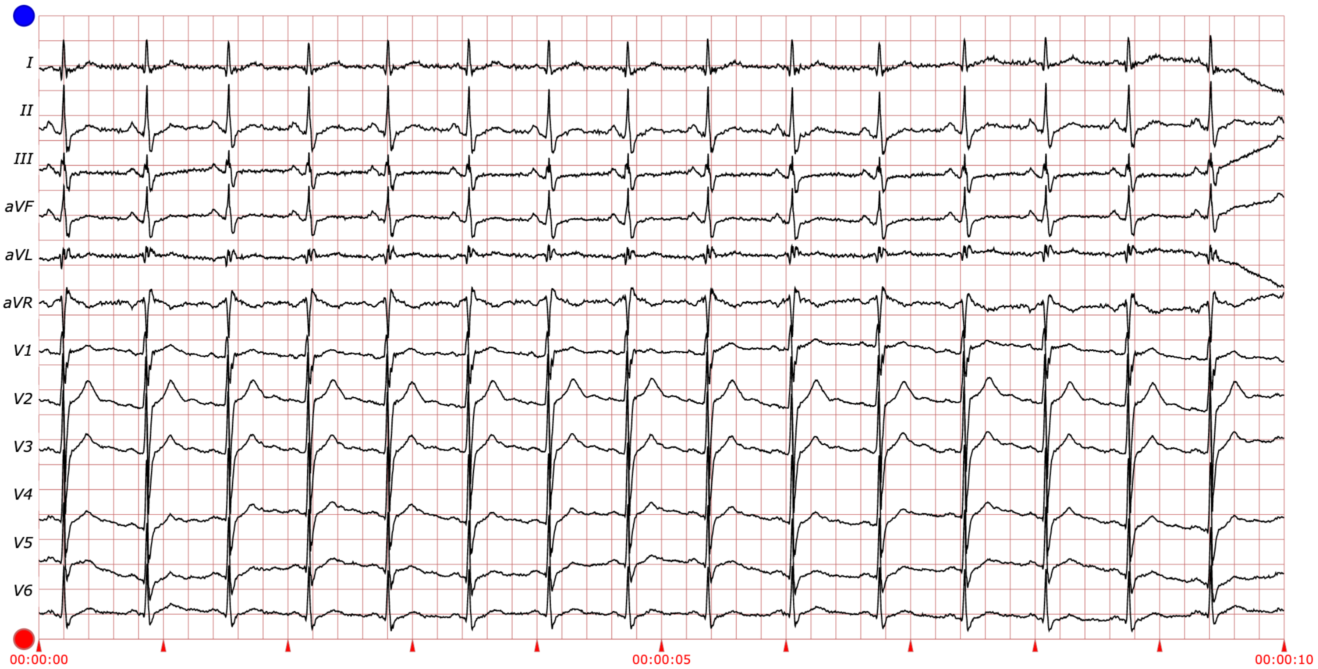

Fig. 1 Normal

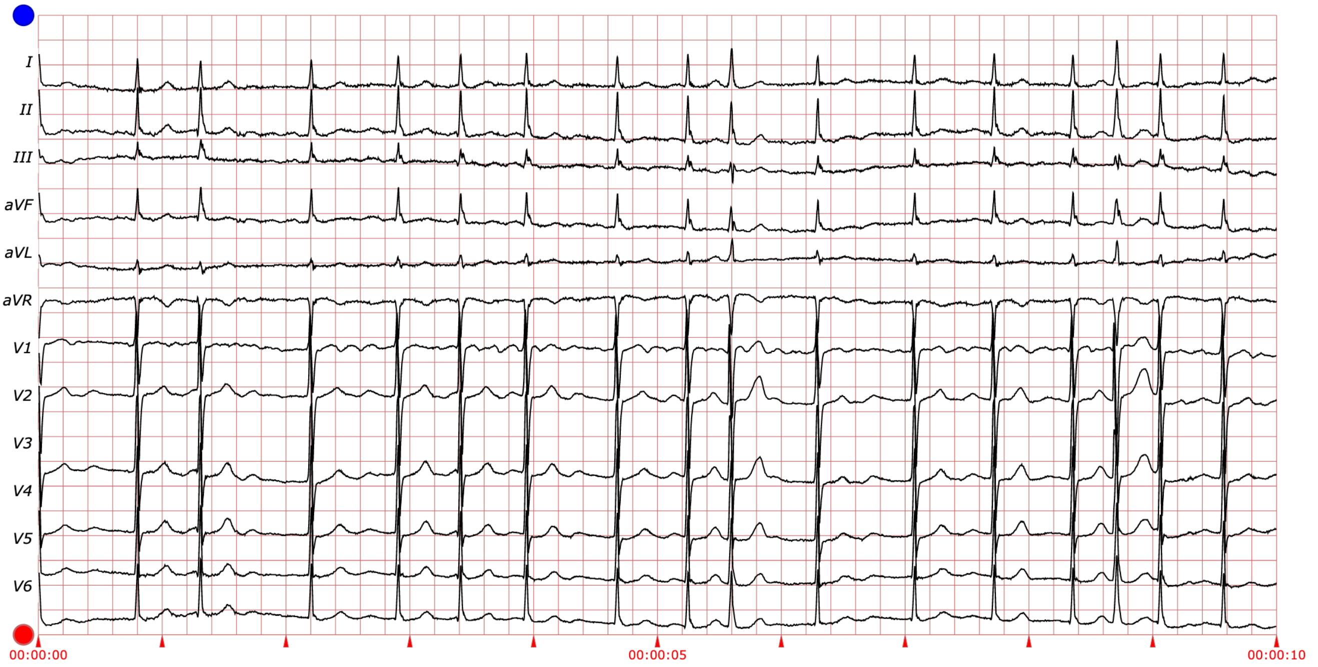

Fig. 2 Atrial fibrillation (AF)

1.3 First-degree atrioventricular node block (FDAVB)

As shown in Figure 3, first-degree atrioventricular block (AV block) is a disease of the electrical conduction system of the heart in which the PR interval is lengthened beyond 0.20 seconds. In first-degree AV block, the impulse conducting from atria to ventricles through the atrioventricular node (AV node) is delayed and travels slower than normal. It has a prevalence in the normal (young adult) population of 0.65-1.1 percent and the incidence is 0.13 per 1000 persons.

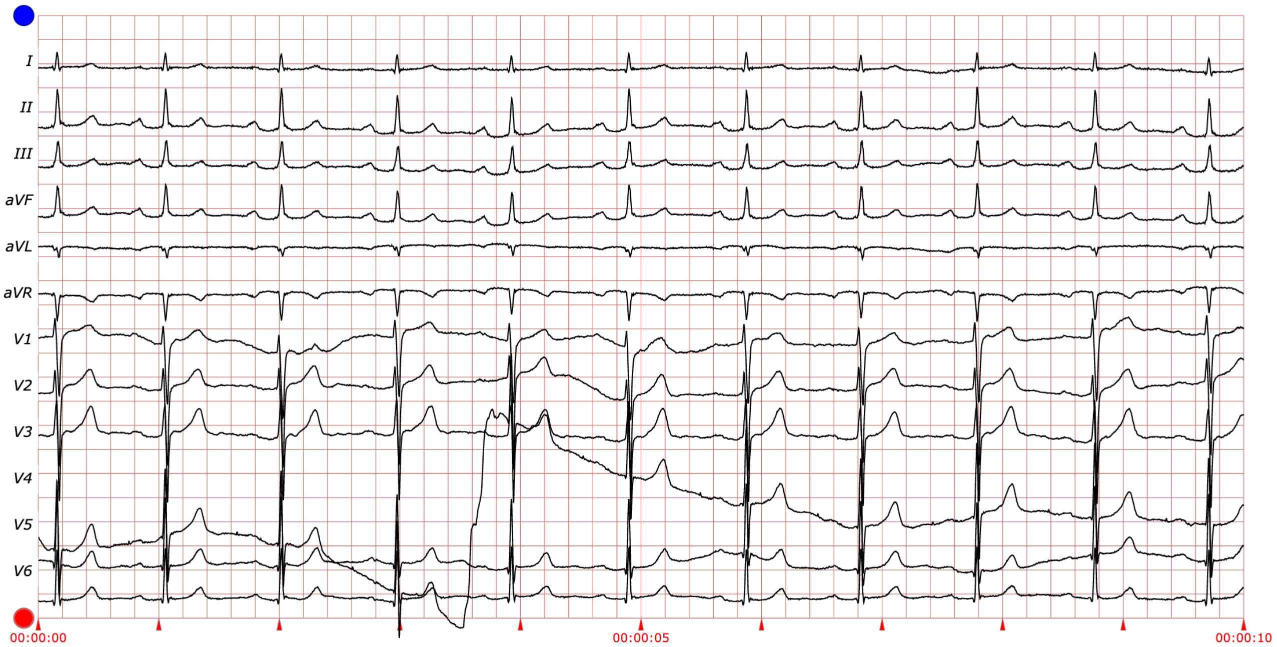

Fig. 3 First-degree atrioventricular node block (FDAVB)

1.4 Right bundle branch block (RBBB)

As shown in Figure 4, right bundle branch block (RBBB) is a heart block in the right bundle branch of the electrical conduction system. During a right bundle branch block, the right ventricle is not directly activated by impulses travelling through the right bundle branch. The left ventricle however, is still normally activated by the left bundle branch. These impulses are then able to travel through the myocardium of the left ventricle to the right ventricle and depolarize the right ventricle this way. As conduction through the myocardium is slower than conduction through the Bundle of His-Purkinje fibers, the QRS complex is seen to be widened. The QRS complex often shows an extra deflection that reflects the rapid depolarization of the left ventricle followed by the slower depolarization of the right ventricle.

1.5 Left anterior fascicular block (LAFB)

As shown in Figure 5, left anterior fascicular block (LAFB) is an abnormal condition of the left ventricle of the heart, related to, but distinguished from, left bundle branch block (LBBB). It is caused by only the anterior half of the left bundle branch being defective. It is manifested on the ECG by left axis deviation. It is much more common than left posterior fascicular block.

1.6 Premature ventricular contractions (PVC)

As shown in Figure 6, premature ventricular contraction (PVC) is a relatively common event where the heartbeat is initiated by Purkinje fibers in the ventricles rather than by the sinoatrial node, the normal heartbeat initiator. PVCs may cause no symptoms at all, but they may also be perceived as a "skipped beat" or felt as palpitations in the chest. Single beat PVC abnormal heart rhythms do not usually pose a danger.

1.7 Premature atrial contraction (PAC)

As shown in Figure 7, Premature atrial contractions (PACs), also known as atrial premature complexes (APC) or atrial premature beats (APB), are a common cardiac dysrhythmia characterized by premature heartbeats originating in the atria. While the sinoatrial node typically regulates the heartbeat during normal sinus rhythm, PACs occur when another region of the atria depolarizes before the sinoatrial node and thus triggers a premature heartbeat. The exact cause of PACs is unclear; while several predisposing conditions exist, PACs commonly occur in healthy young and elderly people. Elderly people that get PACs usually don't need any further attention besides follow ups due to unclear evidence. PACs are often completely asymptomatic and may be noted only with Holter monitoring, but occasionally they can be perceived as a skipped beat or a jolt in the chest. In most cases, no treatment other than

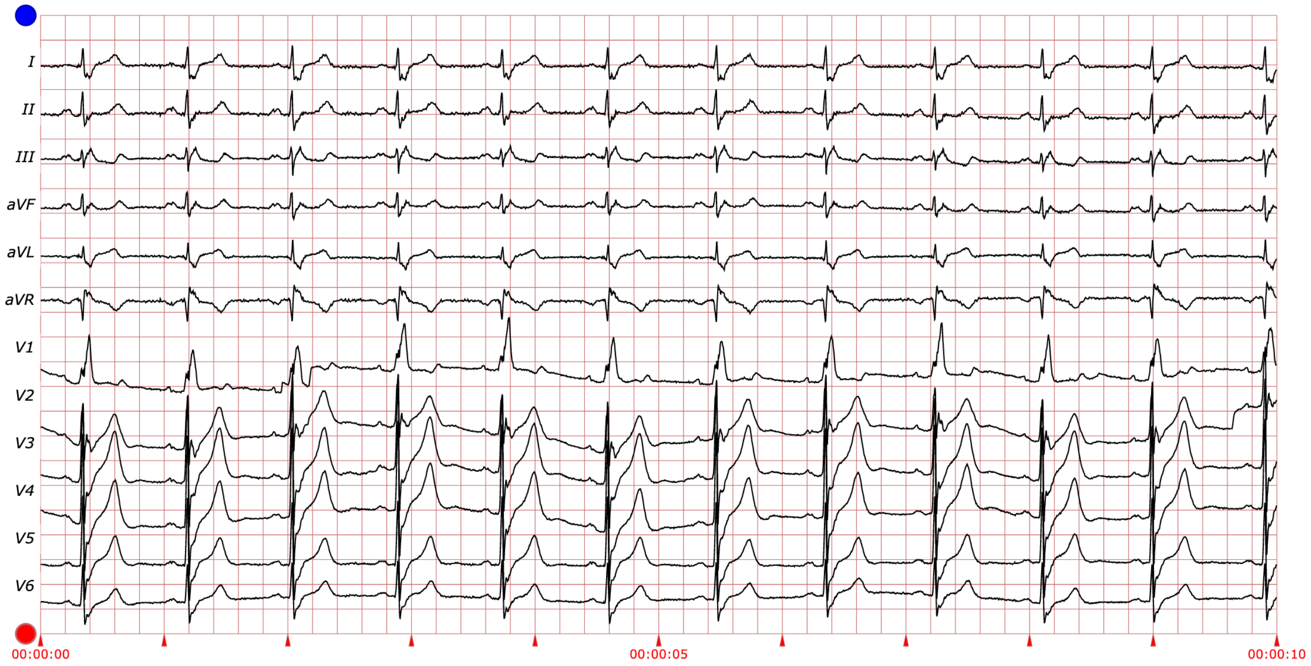

Fig. 4 Right bundle branch block (RBBB)

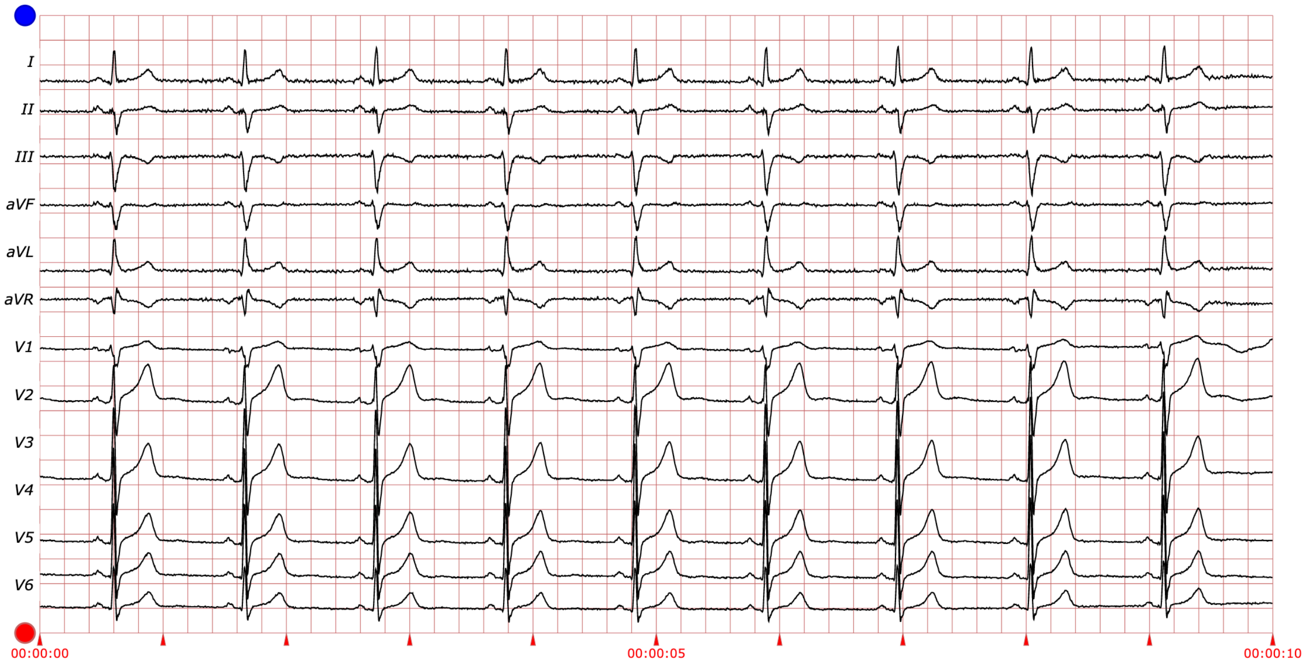

Fig. 5 Left anterior fascicular block (LAFB)

reassurance is needed for PACs, although medications such as beta blockers can reduce the frequency of symptomatic PACs.

1.8 Early repolarization (ER)

As shown in Figure 8, early repolarization also known as early repolarization is found on ECG in about 1 percent of those with chest pain. It is diagnosed based on an elevated J-point / ST elevation where the ST segment is concave up. It is believed to be a normal variant.

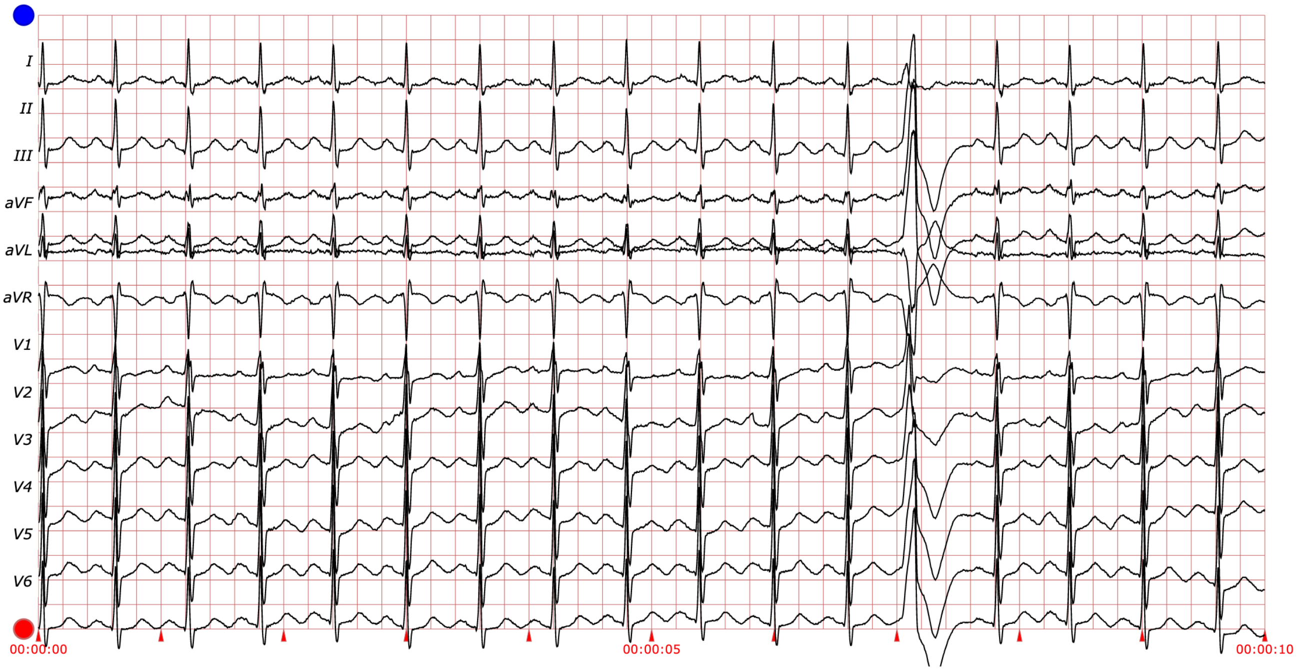

Fig. 6 Premature ventricular contractions (PVC)

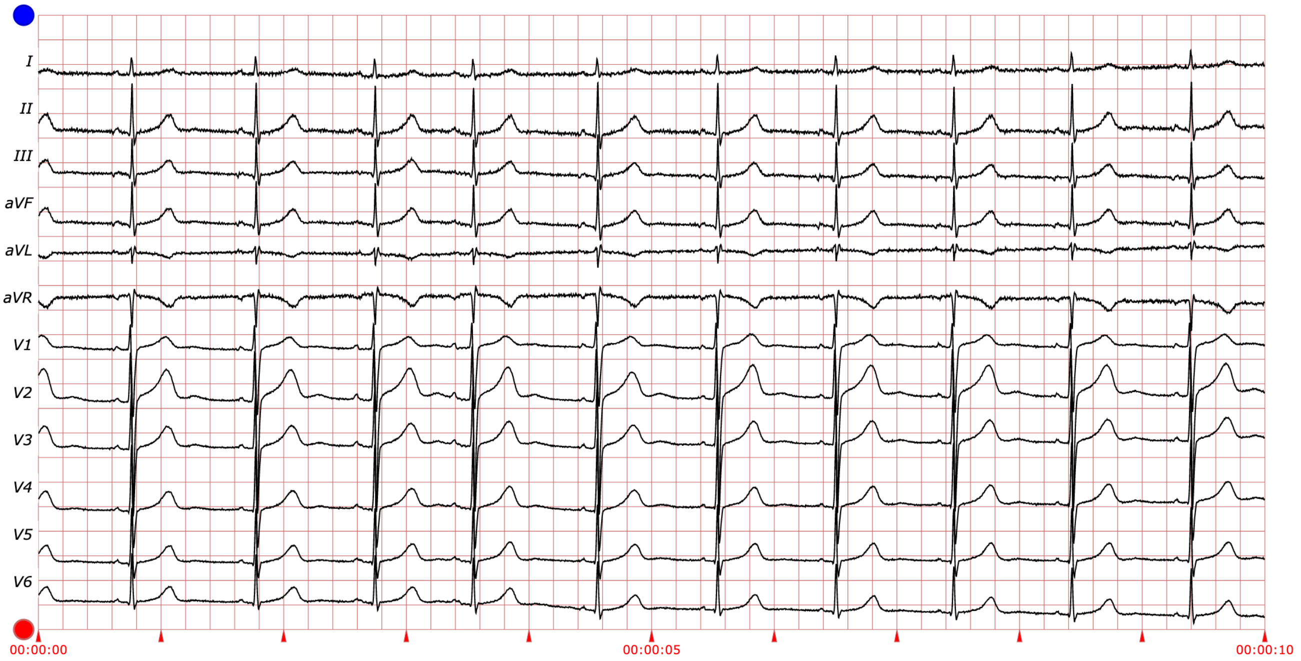

Fig. 7 Premature atrial contraction (PAC)

Benign early repolarization that occurs in the inferior and lateral portion of the heart muscle is associated with ventricular fibrillation. The association, revealed by research performed in the late 2000s, is small.

1.9 T wave change (TWC)

As shown in Figure 9, T wave changes in ECG are used to observe the beating heart, exercise-induced coronary artery dysfunction, acute myocardial ischemia occurs under the endocardium and epicardium of the myocardium, resulting in changes in T wave shape and direction.

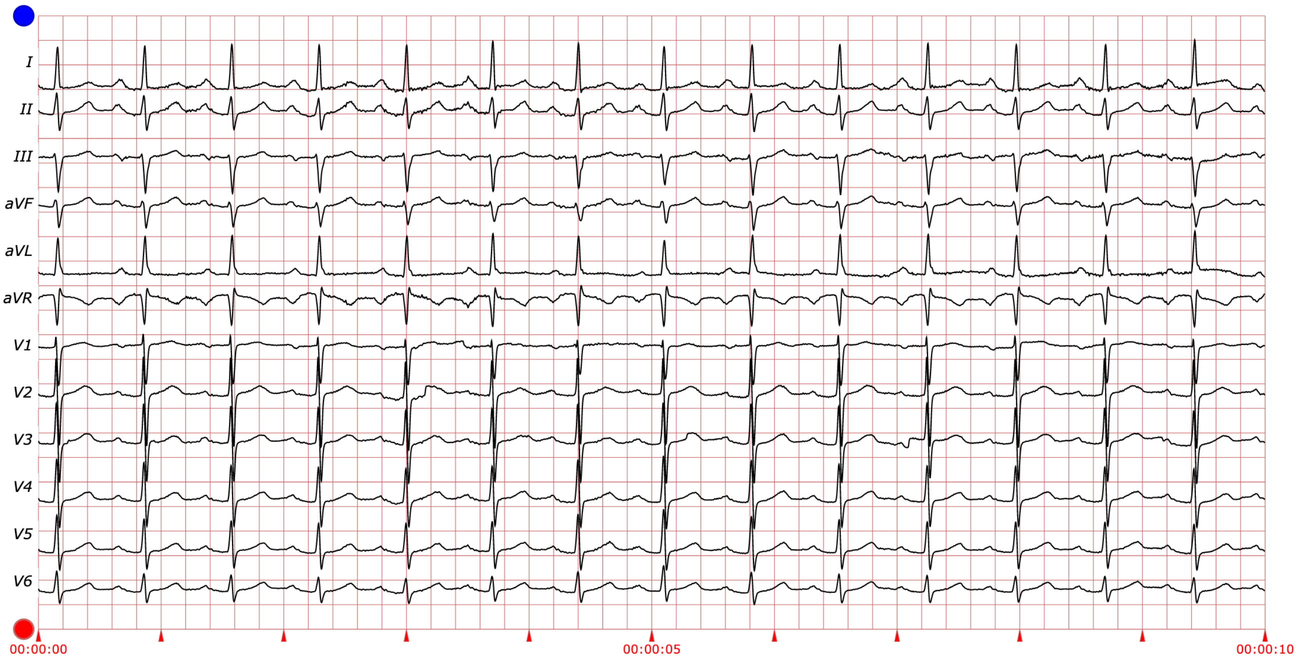

Fig. 8 Early repolarization (ER)

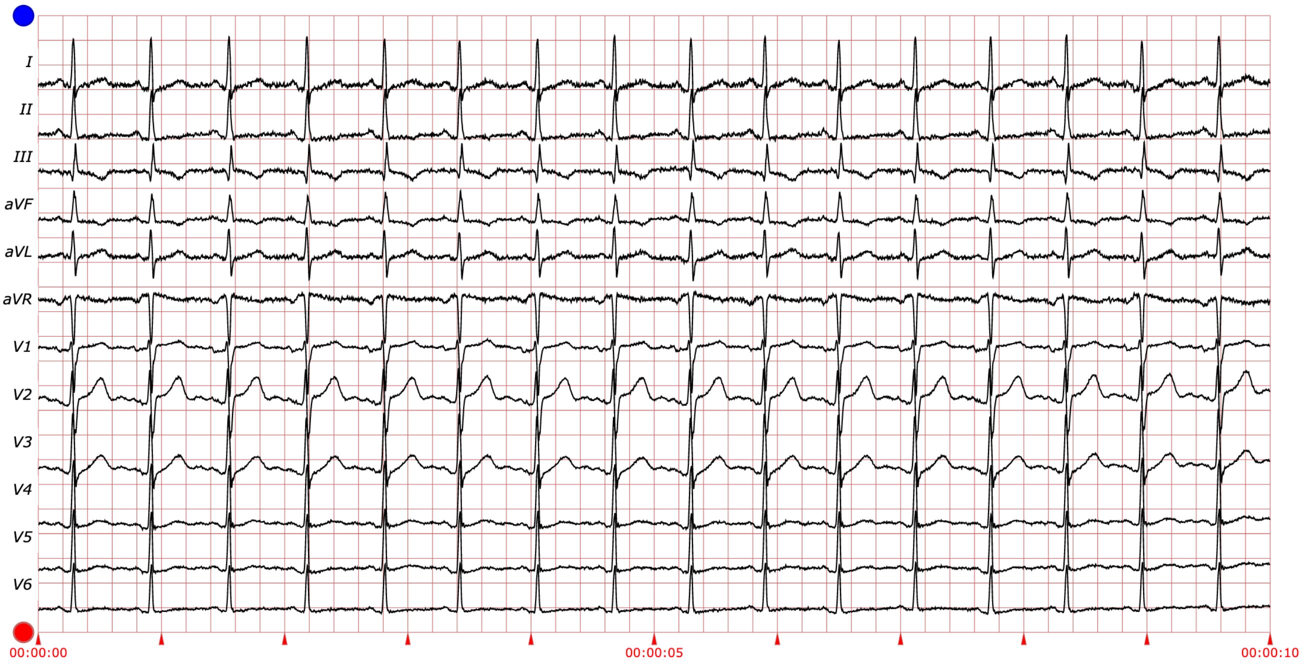

Fig. 9 T wave change (TWC)

1.10 Other ECGs (Others)

Others, which is short for "Other ECGs" here, refers to various types of 12-lead resting ECGs that can not be precisely defined by any of the above mentioned labels. In general, each type belonging to this label has small data size, therefore we combine them togethor to define a type that covers almost all interpretations. The following several types are examples belonging to this label.

1.10.1 Atrial flutter (AFL)

As shown in Figure 10, atrial flutter (AFL) is a common abnormal heart rhythm that starts in the atrial chambers of the heart. When it first occurs, it is usually associated with a fast heart rate and is classified as a type of supraventricular tachycardia. Atrial flutter is characterized by a sudden-onset (usually) regular abnormal heart rhythm on an electrocardiogram (ECG) in which the heart rate is fast. Symptoms may include a feeling of the heart beating too fast, too hard, or skipping beats, chest discomfort, difficulty breathing, a feeling as if one's stomach has dropped, a feeling of being light-headed, or loss of consciousness.

1.10.2 Pre-excitation

As shown in Figure 11, pre-excitation syndrome is an abnormal heart rhythm in which the ventricles of the heart become depolarized too early, which leads to their partial premature contraction.

1.10.3 Myocardial infarction (MI)

As shown in Figure 12, myocardial infarction (MI), also known as a heart attack, occurs when blood flow decreases or stops to a part of the heart, causing damage to the heart muscle. The most common symptom is chest pain or discomfort which may travel into the shoulder, arm, back, neck or jaw. Often it occurs in the center or left side of the chest and lasts for more than a few minutes. The discomfort may occasionally feel like heartburn. Other symptoms may include shortness of breath, nausea, feeling faint, a cold sweat or feeling tired. About 30

2 The Accuracies of each method

The metric accuracy can also reflect how accurate the top-performing methods are. We calcualted the accuracies for each method and found that the discrimination of accuracy is not as good as the $F1$ score.

Table 1 Summary of the Top-Performing 11 Benchmark Methods. All methods are ranked according to their $F1$ scores. The corresponding accuracies are listed.

No.Methods	F1	Accuracy
1	0.882	0.972
2	0.861	0.970
3	0.856	0.972
4	0.853	0.972
5	0.852	0.972
6	0.852	0.972
7	0.848	0.971
8	0.847	0.971
9	0.842	0.971
10	0.841	0.967
11	0.839	0.969

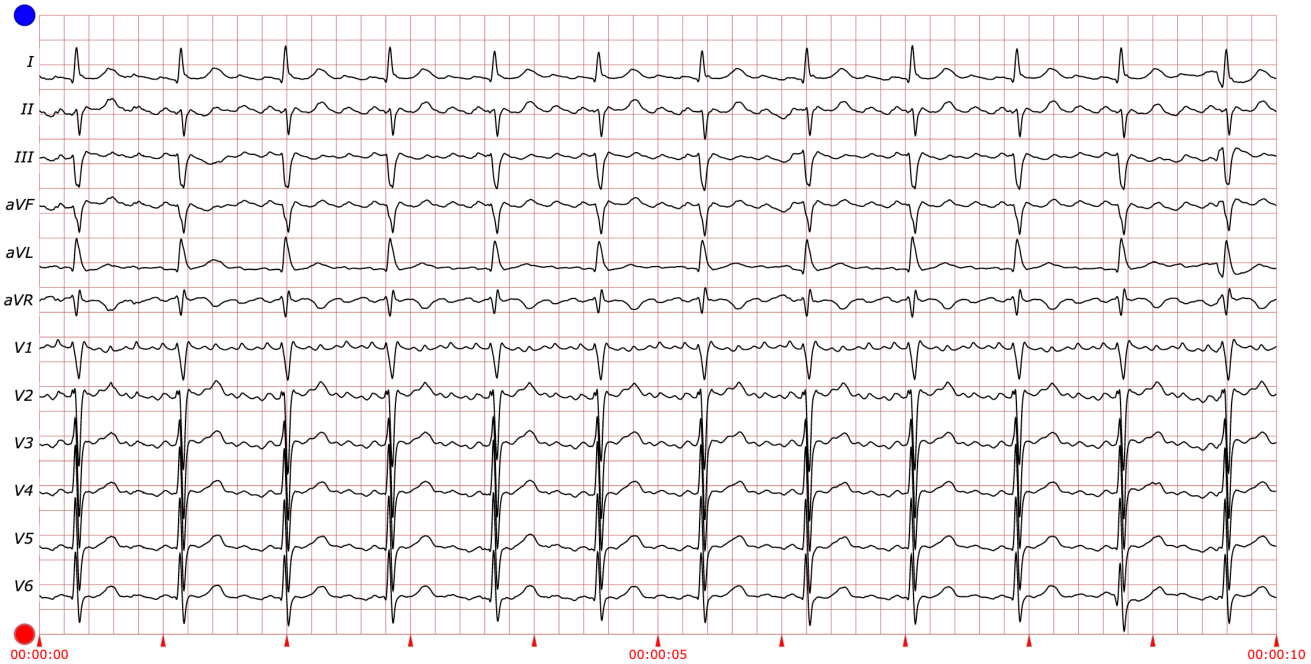

Fig. 10 Atrial flutter (AFL)

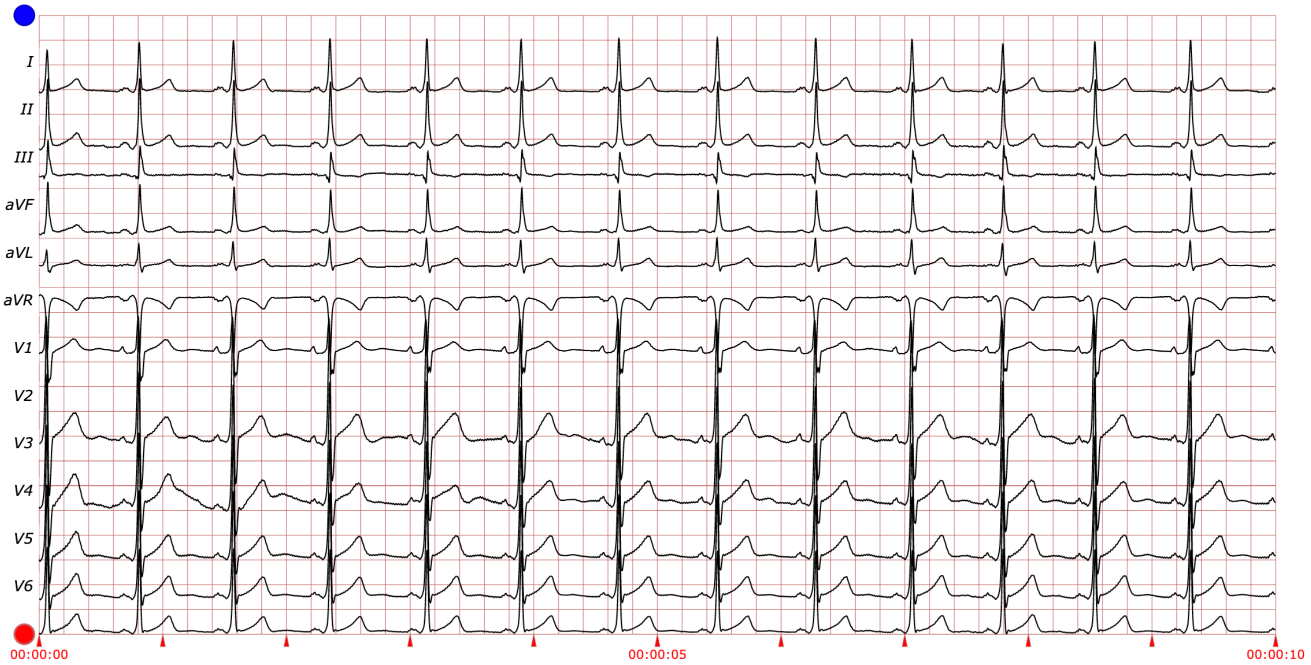

Fig. 11 Pre-excitation

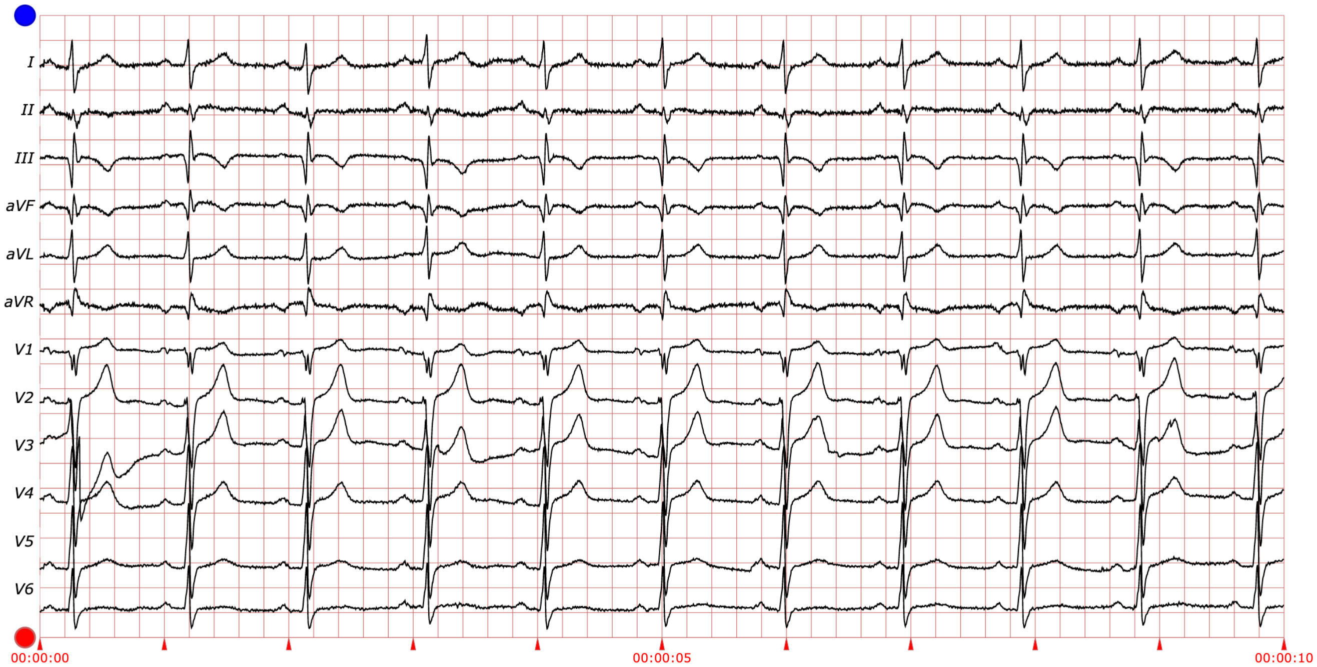

Fig. 12 Myocardial infarction (MI)
